# White matter tracts in first-episode psychosis: A DTI tractography study of the uncinate fasciculus

**DOI:** 10.1016/j.neuroimage.2007.09.012

**Published:** 2008-02-01

**Authors:** Gary Price, Mara Cercignani, Geoffrey J.M. Parker, Daniel R. Altmann, Thomas R.E. Barnes, Gareth J. Barker, Eileen M. Joyce, Maria A. Ron

**Affiliations:** a Institute of Neurology, University College London, Queen Square, London, UK; b Imaging Science and Biomedical Engineering, University of Manchester, UK; c Medical Statistics Unit, London School of Hygiene and Tropical Medicine, London, UK; d Imperial College Faculty of Medicine, Charing Cross Campus, London, UK; e King’s College London, Institute of Psychiatry, Department of Clinical Neuroscience, Centre for Neuroimaging Sciences, UK

## Abstract

A model of disconnectivity involving abnormalities in the cortex and connecting white matter pathways may explain the symptoms and cognitive abnormalities of schizophrenia. Recently, diffusion imaging tractography has made it possible to study white matter pathways in detail, and we present here a study of patients with first-episode psychosis using this technique. We studied the uncinate fasciculus (UF), the largest white matter tract that connects the frontal and temporal lobes, two brain regions significantly implicated in schizophrenia. Nineteen patients with first-episode schizophrenia and 23 controls were studied using a probabilistic tractography algorithm (PICo). Fractional anisotropy (FA) and probability of connection were obtained for every voxel in the tract, and the group means and distributions of these variables were compared. The spread of the FA distribution in the upper tail, as measured by the squared coefficient of variance (SCV), was reduced in the left UF in the patient group, indicating that the number of voxels with high FA values was reduced in the core of the tract and suggesting the presence of changes in fibre alignment and tract coherence in the patient group. The SCV of FA was lower in females across both groups and there was no correlation between the SCV of FA and clinical ratings.

## Introduction

Abnormal brain connectivity has been suggested as the cardinal abnormality in schizophrenia (Friston and Frith, 1995), and a disturbance of connectivity between the frontal and temporal lobes may explain some of its symptoms and cognitive deficits. A disconnectivity model involving both loss of specialized cortical function and damage to connecting pathways is likely (Catani and ffytche, 2005). Imaging studies have repeatedly confirmed the presence of cortical abnormalities, especially in prefrontal and temporal heteromodal cortex (Lawrie and Abukmeil, 1998; Foong et al., 2001; Shenton et al., 2001; Bagary et al., 2003), and synaptic pathology leading to aberrant cortical circuitry has been well documented in neuropathological studies (Harrison and Weinberger, 2005). Abnormal functional connectivity between these two areas has also been demonstrated (Benes, 2000; Friston and Frith, 1995). By comparison, the study of abnormalities in intra- and inter-hemispheric pathways has received much less attention, although the occurrence of schizophrenia-like symptoms has been documented in diseases involving the white matter (Walterfang et al., 2006).

The uncinate fasciculus (UF), the largest of the fronto-temporal connections, is a ventral limbic pathway that originates rostrally in the temporal lobe and terminates in the ventral, medial and orbital parts of the frontal cortex. It connects cortical regions involved in sound and object recognition (superior and inferior temporal gyri) and recognition memory (entorhinal, perirhinal and parahippocampal cortices) with frontal areas implicated in emotion, inhibition and self-regulation. Thus, the UF plays a role in the interaction between cognition and emotion (MacLean, 1952; Barbas, 2000). In humans, lesions involving the UF have resulted in disordered self-regulation, with loss of self-awareness and goal directed behaviour (Levine et al., 1998).

Recent studies have used diffusion tensor imaging (DTI) (Basser et al., 1994) to explore the integrity and orientation of brain tissue in vivo by measuring tissue water diffusion. These studies have measured fractional anisotropy (FA), considered to be an index of white matter integrity, by using a region of interest placed in the UF or by exploring whole brain differences in FA with voxel-based analysis. Kubicki et al. (2002), using a region-of-interest methodology, found higher left than right FA in the UF of healthy controls, with loss of normal asymmetry in patients with schizophrenia. Others (Burns et al., 2003), using voxel-based methodology, have found lower FA in the left UF of patients with chronic schizophrenia compared to controls. Szeszko et al. (2005) have reported a similar left-sided reduction in FA in the white matter of the superior temporal gyrus in a small group of patients with first-episode schizophrenia. This is also in keeping with the findings of reduced magnetization transfer ratio in the fronto-temporal white matter comprising the UF (Bagary et al., 2003).

The information about the direction of diffusion encoded by the eigenvectors of the diffusion tensor has been used in DTI tractography (Mori et al., 1999) to investigate the continuity of axonal orientation between voxels and thus to infer the paths of fibre tracts in three dimensions. DTI tractography has been used in studies of normal subjects (Toosy et al., 2004; Parker and Alexander, 2005; Parker et al., 2005), multiple sclerosis (Wilson et al., 2003), epilepsy (Powell et al., 2004) and stroke (Huang et al., 2005). To date, only a few studies have used DTI tractography to investigate psychiatric illness. Thus, Kanaan et al. (2006), in a group of patients with chronic schizophrenia, reported reduced FA in the genu of the corpus callosum. Another study from the same group (Jones et al., 2006) exploring fronto-temporal connections in a small group of patients with chronic schizophrenia reported no differences in FA between patients and controls in the UF, although FA was reduced in the left superior longitudinal fasciculus in the patient group. Using a probabilistic tractography algorithm (Parker and Alexander, 2003) that takes into account branching of fibres and that assigns a probability of connection to the seed-point to every voxel in the brain, we have previously reported reduced FA in the tracts traversing the genu of the corpus callosum in patients with first-episode psychosis (Price et al., 2007).

Here we present, to our knowledge, the first diffusion MRI tractography study of the uncinate fasciculus in patients with first-episode psychosis in whom chronicity-related factors could be excluded. We used a probabilistic tractography algorithm (PICo; Parker and Alexander, 2003) with a “two-region approach” to study a group of subjects largely consisting of those in whom we had previously reported using tractography of the corpus callosum and using the same DTI data as in our earlier study (Price et al., 2007). We hypothesized that measures of tract coherence (FA) and probability of connection would be significantly reduced in the patient group compared to controls.

## Methods

The patients were recruited as part of a longitudinal study of first-episode psychosis in West London. Initial screening was performed using the WHO Psychosis Screen (Jablensky et al., 2000). Patients were recruited into the study if aged between 16 and 50 years, and if they presented with a psychotic illness for the first time and had received no more than 12 weeks of antipsychotic medication. At the time of screening, initial diagnosis was established by using a structured interview, the diagnostic module of the Diagnostic Interview for Psychosis (DIP), which includes items from the Operational Criteria Checklist for Psychosis (OPCRIT) and the World Health Organization Schedules for Clinical Assessment in Neuropsychiatry (SCAN). From these data, a computerized algorithm generates diagnoses under several classification systems including DSM-IIIR and ICD-10. DSM-IIIR diagnoses were then checked against DSM-IV criteria using OPCRIT for Windows (http://sgdp.iop.kcl.ac.uk/opcrit/).

Nineteen patients were included in the study. Fourteen had a final diagnosis of schizophrenia and five of schizoaffective disorder (1 bipolar, 2 manic, 2 depressed subtypes). The mean age of the patient group was 23.8 years (SD = 6.18; range = 17–38), and the ratio of males to females was 11:8. All patients were receiving antipsychotic medication and two were taking mood stabilizers at the time of the study, and the average duration of drug treatment prior to scanning was 63 days (range 8–153).

Twenty-three healthy subjects (11 males and 12 females) with a mean age of 29.6years (SD = 7.17; range = 16–42) served as controls. Corpus callosum tractography findings in 18 of these patients and 21 of the healthy controls have already been reported (Price et al., 2007).

The range and severity of symptoms was assessed with the Scales for the Assessment of Positive Symptoms and Negative Symptoms (SAPS and SANS; (Andreasen et al., 1981). The age of onset and the duration of untreated psychosis were established using the Symptom Onset in Schizophrenia inventory (Perkins et al., 2000). The patients had been ill for a mean of 27 months before the scanning (SD = 33.31 months, range 1–108) and the mean duration of untreated psychosis was 12 months (SD = 19 months, range 1–72). None of the subjects fulfilled criteria for alcohol or drug dependency (Drake et al., 1990). Handedness in all subjects was assessed using the Annett scale (Annett, 1970).

Exclusion criteria, common to all subjects, were the presence of a medical or neurological illness, including head injury leading to unconsciousness. In addition, controls were excluded from the study if there was a history of psychiatric illness in themselves or their first-degree relatives.

Permission to conduct the study was obtained from Merton, Sutton and Wandsworth, Riverside, and Ealing Research Ethics Committees. All participants gave written informed consent and were paid an honorarium for taking part in the study.

### MRI data acquisition

MRI was performed with a GE Signa 1.5 Tesla scanner (General Electric, Milwaukee, WI, USA), that underwent regular quality control checks, using a standard quadrature head coil. A T_1_-weighted 3D volume was obtained using an inversion recovery spoiled gradient-recalled (IR-SPGR) echo sequence (TE = 5.1 ms, matrix 256 × 256, FoV = 31x16 cm^2^, slice thickness 1.2 mm, TR = 14.3 ms, flip angle = 20°, TI = 450 ms). Diffusion-weighted single-shot echo planar images (DW-EPI) were acquired in the axial plane and gated to the cardiac cycle using a pulse oximeter with a gating scheme optimized for diffusion imaging. Sequence parameters were TE = 96 ms, FoV 22 × 22 cm^2^, matrix = 96 × 96 (reconstructed as 128 × 128 for a final in-plane voxel size of 1.72 mm^2^), slice thickness = 2.3 mm, maximum b value = 1000 smm^− 2^. Gradients for diffusion sensitization were applied in 54 non-collinear directions. Six images with no diffusion weighting (b ≈ 0 s/mm^2^) were also collected for each slice, giving a total of 60 images per slice.

### Diffusion processing and analysis

All diffusion processing and tractography was performed using the CAMINO package (www.cs.ucl.ac.uk/research/medic/camino/; (Cook et al., 2006). The diffusion tensor was estimated for each voxel (Basser et al., 1994) and used to compute FA. We incorporated a model-selection algorithm based on the fit of spherical harmonic series to the diffusion profile (Alexander et al., 2002) to detect the most parsimonious description of diffusion in every voxel. Diffusion within each voxel was classified as isotropic, anisotropic with a single principal direction of diffusion, or anisotropic with more than one direction of diffusion. The single tensor model was subsequently used for voxels characterized by isotropic diffusion or a single direction of diffusion population and the two-tensor model of diffusion (Parker and Alexander, 2003) was used for the remaining voxels.

The tractography method used a probabilistic algorithm (PICo or ‘probabilistic index of connectivity’). This algorithm considers multiple pathways emanating from a seed point or region (i.e. a group of voxels in a region of interest) (Parker and Alexander, 2003; Parker and Alexander, 2005). There is some inherent uncertainty associated with the determination of the principal direction of diffusion due to data noise, and this is accounted for by generating a probability density function (PDF) of fibre alignment from the diffusion model of each voxel (which in this case is either the single or the two-tensor model). PDF provides voxel-wise estimates of confidence in fibre tract alignment. Streamline-based tracking from the seed region was performed using these PDFs and repeated 5000 times in a Monte Carlo fashion, with random perturbations introduced into the PDFs on each repeat. This produced tract maps that estimate the probability of streamline connection of every voxel in the brain to a given seed point or region (Parker and Alexander, 2005). Streamlines were propagated using trilinear interpolation of PDFs, as suggested by Behrens et al.(2003), and were terminated if curvature over the scale of a single voxel exceeded 80 degrees or if the path left the brain. A step size equal to1/10 of the slice thickness was used.

As the UF lies in close proximity to the inferior fronto-occipital and longitudinal fasciculi it is difficult to isolate it from its neighbors, and under these circumstances the so-called “two-region” approach (Conturo et al., 1999) has been shown to be helpful in its reconstruction (Catani et al., 2002). This procedure is more appropriate than simply tracking from a single seed region in delineating the boundaries of the fasciculus and defines, in addition to a seed point or region, a “through” point or region that the reconstructed pathway is constrained to pass through. As the cross-section of the UF is small, the size of the seed region was also small and represented the core of the tract.

**Fig. 1:**
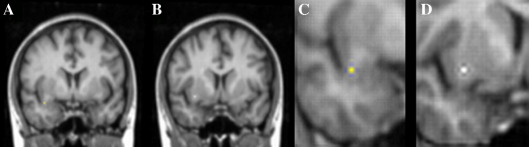
(A) Seed point placed inferiorly in the temporal stem. (B) The “through” region in the lower region of the frontal lobe, at the level of the lateral sulcus and the anterior commissure. Images (C) and (D) are magnified versions of images (A) and (B) respectively.

The seed points in the temporal lobe used for the reconstruction of the UF were placed inferiorly in the temporal stem (the white matter tract between the temporal lobe and frontal lobe), while the “through” region was placed in the frontal lobe, at the level of the lateral sulcus and anterior commissure (Kier et al., 2004) (see Fig. 1). This was the only operator-dependent step in the tractography procedure, and in order to standardize the placement of seed and through points, the non-diffusion-weighted (*b* = 0) EPI scans were registered to the T_1_-weighted images, which were then normalized to a template in Montreal Neurological Institute (MNI) coordinates using FLIRT (FMRIB’s Linear Image Registration Tool; Jenkinson and Smith, 2001). The 12 degrees of freedom affine transformation was optimized using normalized mutual information as a cost function. Two single voxels (one seed and one through-point) belonging to the uncinate fasciculus were identified on the normalized T_1_-weighted image, comparing standard coordinates between subjects to increase confidence in the location. Voxel positions were determined while viewing three mutually orthogonal orientations using MRIcro program (http://www.sph.sc.edu/comd/rorden) and the voxel for each seed and through point were then placed in the axial view. The voxels were then transposed into the space of diffusion-weighted MRI data by inverting the normalization transformation and were superimposed onto FA maps in order to check they were fully included in white matter. Due to the differing voxel size of the images, the transposed seed and “through” regions contained 4 voxels each and were manually checked to ensure partial volume effects were minimized.

**Fig. 2:**
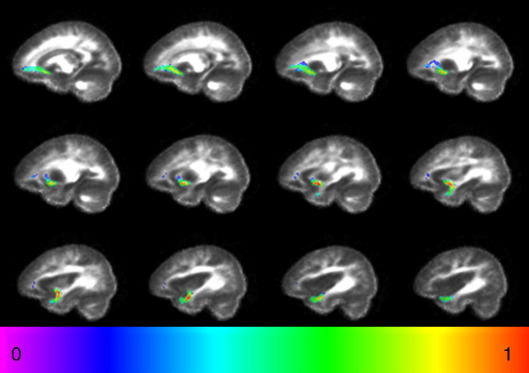
Average reconstruction of the uncinate fasciculus in controls. The map was obtained by averaging a binary mask of the tract for every subject after normalisation. The colour scale indicates the degree of overlap between subjects. ‘0’ represents minimal overlap, ‘1’ represents maximal overlap.

PICo was run from every voxel in the seed region, and only trajectories traversing the through region were retained. The final output was obtained by taking, at each voxel in the brain, the maximum probability value across the probabilistic maps obtained from each seed point. The resultant connectivity maps were thresholded to include only voxels with a probabilistic connection of > 5%. This particular threshold has been used in previous studies (Ciccarelli et al., 2006; Powell et al., 2004) in order to eliminate the voxels most likely to be affected by image noise. The thresholded connectivity map was binarized to obtain an image of the segmented uncinate fasciculus as in our previous study of the corpus callosum (Price et al., 2007). Images were normalized using the same algorithm as above (Jenkinson and Smith, 2001) to a stereotactic space (Montréal Neurological Institute, MNI) using the standard echo planar image template in SPM2 (Wellcome Department of Cognitive Neurology, Institute of Neurology, London, UK) running in MATLAB (MathWorks, Natick, MA, USA) and averaged on a voxel-by-voxel basis to produce a map that displayed of the degree of tract overlap between subjects within each group. An example in the control group, similar in appearance to the patient group, is shown in Fig. 2.

Individual voxel values for FA and probability of connectivity along the tract, as well as the total number of voxels (i.e. a measure of the tract volume), were obtained for all subjects bilaterally. In order to characterize the distribution of FA and probability of connection we calculated, for each subject and separately for the left and right uncinate fasciculi, the mean value and the squared coefficient of variation (SCV), computed as the standard deviation divided by the mean, squared. The SCV is a measure of distribution asymmetry which is sensitive to extreme values on the upper tail (Allison, 1978), when the distribution is skewed. We expected that subtle changes in FA or probability of connection values, such as those we predicted in our patients, would be best detected by the dispersion of FA or probability of connection values in the tails of the data, as this has been shown to be the case in a tractography study of amyotrophic lateral sclerosis (Ciccarelli et al., 2006).

### Statistical analysis

Age, gender and handedness distributions in the two groups were compared using *t*-tests and *χ*^2^ tests. To investigate group differences in mean tract FA, connectivity, volume and SCVs of FA and probability of connection, multiple linear regression analyses were performed with these measures as response (dependent) variables and the following predictors: patient/control indicator, age and gender. The regression analyses of FA, probability of connectivity and SCVs of these measures were also weighted for the number of voxels in each tract contributed by each of the subjects in order to reduce potential bias due to the less precise estimates provided by subjects contributing fewer voxels. Pearson correlations were performed between SCV of FA and global SAPS and SANS scores and duration of illness.

## Results

Patients were younger than controls (mean age in patients = 23.8 years, mean age in controls = 29.6 years). There were no significant differences in gender distribution or handedness between the groups.

There was no significant difference in the FA of the tract between the groups (mean tract FA was 0.319 for the controls and 0.321 for patients). When the five schizoaffective patients were removed from the analysis, the mean FA of the UF in schizophrenic patients and controls did not differ significantly (mean tract FA was 0.319 for the controls and 0.323 for patients).

**Fig. 3:**
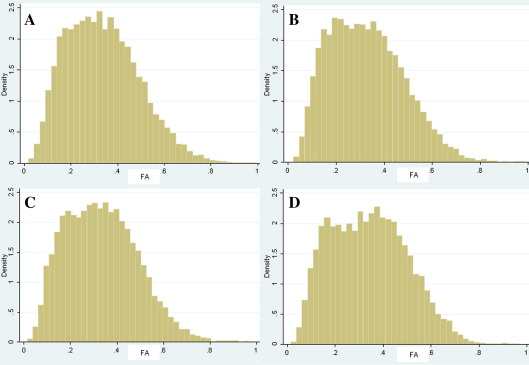
Group histograms of FA values in the left and right uncinate. Histogram ‘A’ is the left uncinate in the patient group. Histogram ‘B’ is the left uncinate in the control group. Histogram ‘C’ is the right uncinate in the patient group. Histogram ‘D’ is the right uncinate in the control group.

The results of the weighted regression analysis of the SCV of FA, incorporating age and gender, revealed a significant difference between patients and controls for the left, but not the right, uncinate fasciculus (coefficient = − 0.035, *t* = − 2.78, *p* = 0.008, 95% CI = − 0.0632 to − 0.009), with lower SCV of FA in the patient group. The SCV probability of connection was not significantly different between the two groups for the left or the right fasciculus. Histograms of FA values for the subject groups for each tract are shown in Fig. 3.

The SCV of FA for the schizophrenia group excluding the five schizoaffective patients, incorporating age and gender, revealed a significant difference between schizophrenic patients and controls for the left, but not the right, UF (coefficient = − 0.046, *t* = − 3.29, *p* = 0.002 95% CI = − 0.0748 to − 0.0176), also with a lower SCV of FA in the patient group.

The SCV of FA was also higher in the left UF in males than in females (difference in mean SCV = 4.271, *p* = 0.042, 95% CI = 0.042 to 8.38), adjusted for age and subject status.

There were no significant correlations between the SCV of FA in the left UF and global SAPS and SANS scores or duration of illness.

## Discussion

Our findings support the evidence for abnormal fronto-temporal connections in patients with first episode psychosis by demonstrating differences in the distribution of FA values (SCV) in the left UF in comparison with a group of normal controls. As the distribution of FA along the tract is skewed to the right, a reduction in the SCV is likely to reflect a smaller upper tail of the distribution in the patient group, and therefore a decrease in the number of voxels with high FA. The different distribution of FA values in our patients, with a reduced number of high FA voxels but no changes in the mean, suggests that the changes responsible for the group differences occur in the core of the tract where the voxels with the highest FA are situated. The neuropathological counterparts of reduced FA are not fully understood. Axonal membranes are thought to be the main determinant of anisotropy in neural tissue, with myelin contributing to a lesser degree. Therefore, alterations in axonal packing density, mean axonal diameter and fibre alignment would result in FA changes. Although complementary, FA and the probability of connection may capture different properties of tract integrity and organization. The probability of connection is a measure sensitive to the length and geometry of a tract. The similar SCV of probability of connection in patients and controls may be due to the fact that in short tracts, such as the UF, measures of probability of connection may not be sufficiently dispersed to result in significant differences between groups. By contrast, SCV of FA may be more sensitive to changes in fibre alignment and tract cohesion likely to be present in our patients.

The only available post-mortem study of the UF in schizophrenia (Highley et al., 2002) described a greater cross-sectional area and number of fibres in the right UF both in normal subjects and patients but failed to detect differences between the two groups. The pathological processes responsible for the changes in FA in the UF observed in our study remain uncertain but are likely to be neurodevelopmental in origin, as suggested by related developmental abnormalities such as the abnormal distribution of interstitial neurons in frontal and temporal regions (Eastwood and Harrison, 2003; Eastwood et al., 2006) where the fasciculus originates, and abnormalities in genes encoding myelin (Hakak et al., 2001). Our results are also in keeping with those of our previous corpus callosum tractography study in the same patients (Price et al., 2007), where changes in FA also occurred in the core of the genu with a greater variance of tract volumes in the patient group. We interpreted this combination as an indication of greater branching (i.e. less coherence or alignment) in the core of the tract. The findings of a gender difference, with greater tract coherence in males on the left UF irrespective of diagnostic group, echo our previous findings (Price et al., 2007) in the same subjects of higher FA in males in the genu of the corpus callosum. Gender dimorphism and maturational differences are likely to be relevant in explaining this finding.

The clinical significance of changes in FA in the UF is still uncertain. The cholinergic pathways contained within the UF innervate frontal and temporal cortical regions (Selden et al., 1998) mediating information processing (Mesulam, 1981), and changes in these pathways may contribute to the symptoms of psychosis and to cognitive changes, e.g. memory deficits (Nestor et al., 2004). The lack of significant correlations between DTI tractography and symptoms may be due to our small sample size; alternatively the expression of the symptoms we measured may be linked to abnormalities in other brain areas. A more likely explanation, however, is the instability of clinical ratings, as symptoms tend to respond to medication while the DTI measures are more likely to remain stable over time. This is also in keeping with the possibility that abnormalities described here may be markers of vulnerability to psychosis and therefore unrelated to symptom severity.

The uncinate fasciculus is the largest of the fronto-temporal connections, which also include the cingulum bundle and the superior longitudinal and arcuate fasciculi. Several DTI studies using voxel-based methodologies have examined the UF in patients with schizophrenia. Reduction of FA in the left UF has been reported in patients with chronic (Burns et al., 2003) and first-episode schizophrenia (Szeszko et al., 2005) and other studies have reported FA reduction in other fronto-temporal connections, but not in the UF. Thus, Kubicki et al. (2005) reported FA reduction in the cingulum bundle bilaterally and in the left arcuate fasciculus, and Shergill et al. (2007) described bilateral FA reductions in the superior longitudinal fasciculus, but not in the white matter corresponding to the UF, in patients with chronic schizophrenia compared with controls. The discrepancies between these studies may be due to differences in methodology (e.g. slice thickness), but the inherent difficulties of the transfer of the diffusion tensor to standard space may also be relevant (Park et al., 2003). Using tractography, Jones et al. (2005), in a small group of patients with very-late-onset schizophrenia-like psychosis, failed to find FA changes in the reconstructed UF and superior longitudinal fasciculus. In a more recent small study (Jones et al., 2006), using a methodology similar to our own, the same investigators found FA reductions in the superior longitudinal fasciculus in the patient group, but not in the UF. The sample size of this study may not have been large enough to detect the subtle differences, and means rather than SCVs were used for group comparison.

The study of patients with first-episode psychosis minimizes the effects of chronicity and lengthy exposure to medication, but such patients are, by definition, diagnostically heterogeneous and this could be seen as a shortcoming of this study. However, when patients with schizoaffective disorder were excluded the FA changes remained significant suggesting that these abnormalities are present in schizophrenia at disease onset. The small number of patients with schizoaffective illness did not allow further comparisons between the patient subgroups. However, there is evidence to suggest that changes in FA are present in other psychosis. Thus, Bachmann et al. (2003) reported changes in FA in the corpus callosum in a heterogeneous group of first-episode psychosis patients, and others (Brambilla et al., 2003, 2004; Szeszko et al., 2005) have described DTI changes in the white matter of patients with bipolar disorder, including those in their first episode of mania (Adler et al., 2006). There is also evidence that oligodendrocyte and myelination genes may be downregulated both in schizophrenic and affective psychosis (Tkachev et al., 2003; Iwamoto et al., 2005). A further shortcoming of our study is the small sample size, which may have made it difficult to detect changes in other DTI parameters.

A strength of our study is the fact that it was possible to isolate and examine most of the UF rather than a region of interest. The use of summary statistics besides the mean (i.e. the SCV) for FA and probability of connection is a further strength, as these statistics are appropriate when comparing differences between sets of skewed data and are complementary when exploring subtle changes in connecvity (Allison, 1978; Ciccarelli et al., 2006). Although our groups were not matched in age – a factor which may influence the FA values of frontal connections (Jones et al., 2006) – we did incorporate age in the statistical analysis to control for its possible effects.

Antipsychotic medication may have played a role in reducing FA, although it seems unlikely that medication effects could fully account for our findings in first-episode patients who had been exposed to medication for short periods. Moreover, others (Flynn et al., 2003) have found no correlation between the presence of white matter abnormalities in first-episode psychosis and exposure to antipsychotic medication. The possible effect of mood stabilizers in explaining our results is likely to be negligible, as they were used in only two patients.

Our study demonstrates that tractography is a useful tool to study structural connectivity in psychiatric disease and suggests that abnormalities in white matter tracts are present early in the disease. Future studies are needed to replicate these findings and to determine whether the abnormalities described here are progressive.
